# Assessment of *Campylobacter fetus* subsp. *venerealis* molecular diagnosis using clinical samples of bulls

**DOI:** 10.1186/s12917-020-02634-7

**Published:** 2020-10-29

**Authors:** Marta Filipa Silva, Ana Duarte, Gonçalo Pereira, Luísa Mateus, Luís Lopes-da-Costa, Elisabete Silva

**Affiliations:** CIISA - Centro de Investigação Interdisciplinar em Sanidade Animal, Faculdade de Medicina Veterinária, Universidade de Lisboa, Avenida da Universidade Técnica, 1300-477 Lisbon, Portugal

**Keywords:** Bovine genital Campylobacteriosis, *Campylobacter fetus* subsp. *venerealis*, Molecular diagnostics

## Abstract

**Background:**

*Campylobacter fetus* subsp. *venerealis* (*Cfv*) is the pathogen responsible for Bovine Genital Campylobacteriosis (BGC), a venereal disease of cattle associated with impaired reproductive performance. Although several PCR assays were developed to identify this pathogen, most of them are still poorly evaluated in clinical samples. This study evaluated real-time PCR assays for *Cfv* detection in preputial samples of bulls (*n* = 308).

**Results:**

The detection at the subspecies level (*Cfv*) compared four assays: two targeting ISCfe1 and two targeting *parA* gene. The detection at the species level (*C. fetus*) considered an assay targeting the *nahE* gene and a commercial kit for *C. fetus* identification. At the subspecies level, assays directed either to different targets (*parA* and ISCfe1), or to the same target (ISCfe1 or *parA*), showed a high percentage of disagreeing results. All samples positive at the subspecies level (*n* = 169) were negative in *C. fetus* detection assays, which strongly suggests the horizontal gene transfer of ISCfe1 and *parA* to other bacterial species. This was confirmed by microbiological isolation of three *Campylobacter portucalensis* strains responsible for false positive results. Sequences with a high level of identity with ISCfe1 and *parA* gene of *Cfv* were identified in *C. portucalensis* genome.

**Conclusions:**

Overall, this study reveals that PCR assays solely directed to a subspecies target originate a high rate of false positive results, due to the presence of *parA* and ISCfe1 homologous sequences in other bacterial species, namely of the *genus Campylobacter*. Although the specificity of these methods may be higher if applied to bulls from herds with clinical features of BGC or in other geographical regions, current PCR diagnosis should couple subspecies and species targets, and further research must be envisaged to identify *Cfv* specific molecular targets.

**Supplementary Information:**

The online version contains supplementary material available at 10.1186/s12917-020-02634-7.

## Background

Bovine Genital Campylobacteriosis (BGC) is a venereal disease of cattle, caused by the bacterial pathogen *Campylobacter fetus* subsp. *venerealis* (*Cfv*), responsible for reproductive failure and significant economic losses, mainly in beef herds where natural breeding prevails [1, 2]. Bulls are asymptomatic carriers, harbouring *Cfv* in the preputial crypts, and infect females during breeding [3]. By contrast, *Cfv* infection in females induces endometritis, early embryonic death and abortion [2]. An accurate diagnosis of BGC is essential for implementation of disease control programs and international trade of bulls and semen [4]. However, BGC diagnosis is hindered by the two cattle-associated *Campylobacter fetus* subspecies, *C. fetus* subsp. *fetus* (*Cff*) and *Cfv,* with distinct niche preferences but with similar genotypic and phenotypic characteristics [5, 6]. Although *Cff* inhabits cattle intestinal tract, it can be occasionally recovered from bovine preputial samples and it is responsible for sporadic cases of abortion [1, 7]. The OIE recommended method for BGC diagnosis is microbiological isolation of *C. fetus*, followed by the 1% glycine tolerance test, for which *Cfv* is intolerant and *Cff* is tolerant [2]. Due to the fastidious growth of *Cfv* and potential overgrowth of other microorganisms, microbiologic techniques are laborious, time-consuming and associated to poor sensitivity [1]. Additionally, *Cfv* can acquire glycine tolerance by mutation or transduction mechanisms being misidentified as *Cff* [8]. Therefore, molecular biology techniques have evolved as attractive tools for diagnosis of BGC. At the species level, real-time PCR assays to detect the *nahE* gene of *C. fetus* were developed and validated, showing high specificity and sensitivity [9, 10]. However, the identification at the subspecies level is hampered by the high genetic similarity of *Cfv* and *Cff* [11]. Nevertheless, several PCR assays have been described to identify *Cfv* [12–15]. The most used molecular targets for *Cfv* identification are the *parA* gene and the ISCfe1 insertion element [9, 10, 13, 14]. Assays based on ISCfe1 detection are described as more sensitive than *parA*-based assays to identify *Cfv* isolates [9, 10]. Although real-time PCR assays targeting the *parA* gene were evaluated in clinical samples [13, 16–18], available data is insufficient to allow its routine use for diagnosis of BGC. Assays based on ISCfe1 detection were not yet assessed in clinical samples despite showing promising results on *Cfv* isolates [9, 10]. Therefore, the diagnostic value of PCR assays for bull clinical samples has not been elucidated. This is a relevant clinical issue because bull testing is the most appropriate prophylactic measure for BGC control. This study evaluated different real-time PCR assays for *Cfv* identification on bull preputial samples in order to assess their suitability for BGC diagnosis.

## Results

### Performance of the real-time PCR assays

The performance of the real-time PCR assays was evaluated using DNA from preputial samples spiked with *Cfv* genome copies. All assays were able to detect 10 to 10^5^ *Cfv* genome copies within 40 cycles of amplification and considering a positivity threshold set to Ct ≤ 35, were able to detect 100 *Cfv* genome copies. The efficiency (Table 1) was within the acceptable range of 90–110% [19] and a linear relationship between the cycle threshold (Ct) value and the log of copy number was evidenced by an *r*^2^ ≥ 0.98. The intra- and inter-assay coefficients of variation were less than 5%, as shown in Table 1.

**Table 1:** Performance parameters of the real-time PCR assays

Assay	Slope	Y-Intercept	***r***^**2**^	E (%)	Intra-assay CV (%)	Inter-assay CV (%)
ISC-A	−3.3732	36.472	0.99	97.90	≤ 1.22	≤ 0.71
ISC-B	−3.3193	37.285	0.99	100.1	≤ 1.41	≤ 0.98
parA-A	−3.1270	38.134	0.98	108.8	≤ 0.81	≤ 1.37
parA-B	−3.3723	40.008	0.99	97.94	≤ 2.01	≤ 1.05
*nahE*	−3.4440	37.773	0.99	95.20	≤ 1.88	≤ 0.61

*E* Efficiency of amplification, *CV* coefficient of variation.

### Detection of *Cfv* and *C. fetus* molecular targets in preputial samples

Four different assays for *Cfv* identification were tested in clinical samples with unknown BGC sanitary status, targeting the ISCfe1 sequence (ISC-A and ISC-B assays) and the *parA* gene (parA-A and parA-B assays). The agreement between parA-A and ISC-A assays was evaluated in all the 308 bovine preputial samples. The parA-A assay gave a lower number of positive results (*n* = 78, 25.3%) than the ISC-A assay (*n* = 155, 50.3%) (*P* < 0.001). This originated a high percentage of disagreeing results (34.1%, Kappa = 0.32), mainly represented by ISCfe1 positive and *parA* negative samples (Table 2).

**Table 2:** Agreement between parA-A and ISC-A assays

parA-A	ISC-A		Total
	Positive	Negative	
**Positive**	64 (20.8%)	14 (4.5%)	78 (25.3%)
**Negative**	91 (29.5%)	139 (45.1%)	230 (74.7%)
**Total**	155 (50.3%)	153 (49.7%)	308

**Fig. 1:**
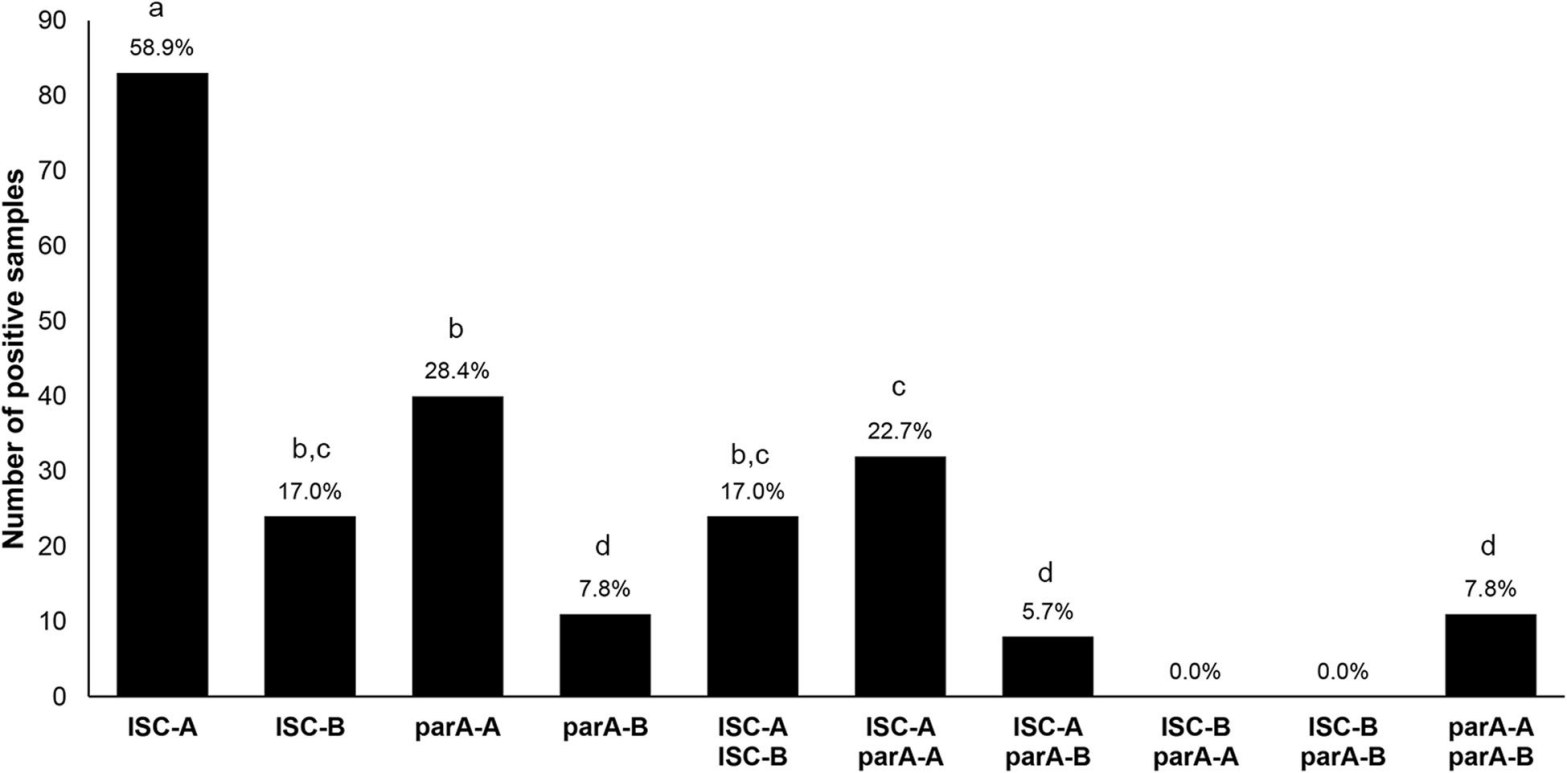
Distribution of positive results identified by each assay and double combinations of assays (*n* = 141). The percentage of positive results is displayed above the columns. Different letters above columns indicate statistically significant differences (*P* < 0.05)

The conservation of *parA* and ISCfe1 sequences was evaluated on a subset of 141 samples, comparing results obtained with assays targeting two different regions of each nucleotide sequence (ISC-A/ISC-B and parA-A/parA-B assays). The parA-A and parA-B assays provided 28.4 and 7.8% positive results, respectively, resulting in a high percentage of disagreeing results (20.6%; Kappa = 0.35) (Additional file 1). Likewise, the ISC-A and ISC-B assays originated 58.9 and 17.0% positive results, respectively, also resulting in a high percentage of disagreeing results (41.8%; Kappa = 0.25) (Additional file 1). These differences observed between assays directed towards the same target are statistically significant (*P* < 0.01). The number and percentage of positive samples detected by each assay, or double combination is shown in Fig. 1, for all samples tested by the four assays (*n* = 141). Results obtained by different assays were highly inconsistent. ParA-B assay provided the lowest number of positive samples (*n* = 11), while ISC-A assay detected the highest number of positive samples (*n* = 83). In contrast, there were no samples tested simultaneously positive by ISC-B and *parA* detection assays.

The presence of genes from the *Cfv*-associated genomic island (GI), which may contain the *parA-A* gene, was evaluated by PCR. Results showed that genes of this GI (*fic1*, *fic2*, *virB9*, *virB11*) were detected in 95% (*n* = 18) of the parA-A positive samples (Table 3), and a significant association was found between the presence of the *parA* gene and *C. fetus* GI genes (φ = 0.873, *P* < 0.001).

**Table 3:** Comparison of results obtained with the parA-A assay and amplification of *C. fetus* specific GI genes

parA-A	GI-associated genes		Total
	Positive	Negative	
**Positive**	18 (36.7%)	1 (2.0%)	19 (38.8%)
**Negative**	2 (4.1%)	28 (57.1%)	30 (61.2%)
**Total**	20 (40.8%)	29 (59.1%)	49

GI - genomic island; GI-associated genes - *fic1*, *fic2*, *virB9*, *virB11*

To evaluate the specificity of the *parA* gene and ISCfe1 as *Cfv* diagnostic targets, samples tested positive for at least one of these targets were also tested using a real-time PCR assay to detect the *C. fetus* specific *nahE* gene and a commercial diagnostic kit (VetMAX C. fetus kit) approved *for C. fetus* detection in DNA isolated from clinical samples. These assays detect targets common to both *C. fetus* subspecies (*Cff* and *Cfv)*. All samples (*n* = 169) that tested positive to *Cfv* specific targets (*parA* gene and/or ISCfe1) were negative in *C. fetus* detection assays.

### Impairment of *Cfv* detection in clinical samples by *Campylobacter portucalensis*

**Fig. 2:**
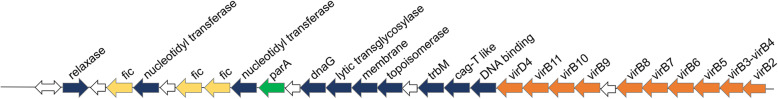
Schematic representation of the genomic island identified in *C. portucalensis* FMV-PI01. Orange arrows represent T4SS genes. White arrows indicate genes with unknown function

To investigate the cause of false positive results, microbiological culture and isolation of *Campylobacter* spp. was performed in three samples positive to *parA* and/or ISCfe1 and negative in *C. fetus* detection assays. Three strains identified biochemically and by 16S rRNA gene sequencing as *Campylobacter portucalensis* [20] were isolated from these samples. These strains were confirmed to be responsible for false positive results in the above *Cfv* detection assays. Whole genome sequencing (WGS) data of the type strain *C. portucalensis* FMV-PI01^T^ (NCBI accession no: VWSJ00000000) allowed the identification of the *parA* gene (98.1% identity with sequence from *Cfv* strain WBT011/99) and of an insertion sequence highly similar to ISCfe1 (93.5% identity with sequence from *Cfv* strain zaf3). The WGS of FMV-PI01 also showed that *parA* gene is located in a GI, which is highly homologous to the *Cfv-*associated GI with 97.42% of identity in 80% of the sequence. The arrangement of *parA*, *fic* and T4SS genes in the GI is schematically represented in Fig. 2.

Two of the *C. portucalensis* strains harbouring *Cfv* molecular markers, tested positive for both ISCfe1 and *parA* gene by ISC-A and parA-A assays, and GI-associated genes, whereas one strain tested positive for ISC-A and negative for parA-A and GI-associated genes.

## Discussion

The analytical performance of the assays used in this study was evaluated using preputial samples spiked with *Cfv* DNA. Assays directed to species and subspecies targets showed similar analytical sensitivities, detecting 10^1^ to 10^2^ genome copies of *Cfv* NCTC 10354. All assays provided reproducible results in the same experiment and between experiments, as evidenced by the coefficient of variation (CV) below 5%. Overall, the results showed a good analytical performance of real-time PCR assays and indicate their suitability for detection of *Cfv* in the bovine preputial sample matrix.

A considerable disagreement was observed between results obtained with parA-A and ISC-A assays. This was also observed for assays targeting the same molecular target (ISC-A/ISC-B and parA-A/parA-B), which suggests divergences in the nucleotide sequences of *parA* and ISCfe1, specifically in primer and probe binding sites. Moreover, in a considerable number of samples only one of the molecular targets was detected. This finding evidences a sensitivity and/or specificity failure of one or both detection assays. Previous studies showed that the *parA*-based PCR assays have a lower sensitivity when compared with assays targeting ISCfe1 for the identification of *Cfv* isolates [9, 10]. In fact, *parA* sequence variation may potentially hinder *parA*-based diagnostic methods [16, 21]. On the other hand, at least two different ISCfe1 sequences were found in *Cfv* strains, sharing 98.7% sequence homology [10]. Therefore, inconsistent results obtained by assays directed towards the same target may result from divergences in the nucleotide sequences.

Samples positive for subspecies specific targets revealed negative in the *C. fetus* detection assays. These samples were expected to be positive for *C. fetus* detection, since the species-specific assays are based on targets common to both *C. fetus* subspecies (*Cff* and *Cfv*). The *nahE* detection assay showed an analytical sensitivity comparable to that obtained with the subspecies detection assays, indicating that the absence of amplification is not related with differences in the analytical sensitivity. Moreover, although all results obtained with the VetMAX *C. fetus* kit were negative, the amplification of an internal positive control allowed to validate results. These findings strongly suggest that subspecies targets were horizontally transferred to other bacterial species. Although the *parA* gene is still used for *Cfv* identification, the horizontal gene transfer of *parA* to *C. hyointestinalis* isolates and *Cff* strains was reported [22, 23]. Also, *parA* detection assays identified putative positive *Cfv* bulls in herds without impairment of the reproductive performance [24] and in virgin bulls [17, 25]. Indeed, the *parA* gene can be found in mobile genetic elements, namely in a GI almost exclusive of *Cfv* [26, 27], in the chromosome and in extra-chromosomal plasmids [21, 27]. On the other hand, genome sequencing data recently deposited in the NCBI database revealed that ISCfe1 can be found in plasmids (e.g. acc. no. CP043436.1), which may facilitate the horizontal transfer of this sequence to non-*Cfv* microorganisms. As a transposable element, ISCfe1 may spread in the genome, thus justifying the variable number of ISCfe1 copies in different *Cfv* strains [14].

Although the possibility of the above targets being transferred horizontally has already been described or suggested, there is no indication in the literature that assays based on these targets can lead to such a high rate of false positive results as those found in this study. In fact, these targets are still used as sole molecular targets for *Cfv* detection, as evidenced in recent studies [28–30]. The rate of false positive results may depend on the geographical region, since previous studies in Canada [18] or Brazil [28, 29] reported lower rates of positive results with *parA*-based assays.

*C. portucalensis,* which is an inhabitant of the bull’s prepuce [20], was identified as a cause of false positive results. The high identity between the sequences found in *C. portucalensis* and ISCfe1 and *parA* sequences of *Cfv* suggests the horizontal transfer of these molecular targets. Although *C. portucalensis* and *Cfv* are not phylogenetically close species [20], the sharing of the same niche by *Cfv* and *C. portucalensis* may have facilitated the horizontal gene transfer of *parA* and ISCfe1. The genomic island associated to *Cfv,* in which the *parA* gene is included, was found in the genome of *C. portucalensis*, supporting the results of *parA* positive samples, with amplification of GI genes (*fic1, fic2, virB11, virB9*) and without amplification of *C. fetus* specific molecular targets. One of the *C. portucalensis* strains is positive for ISCfe1, without amplification of GI genes or *parA*. This finding suggests an independent horizontal gene transfer of *parA* and ISCfe1 and may justify the high percentage of samples positive in ISC-A assay and negative in parA-A assay (*n* = 91, 29.5%). The high similarity between the sequences found in *C. portucalensis* and ISCfe1 and *parA* gene sequences of *Cfv* is enough to impair the accuracy of molecular diagnostic methods based on these targets’ detection.

Overall, this study evidenced that ISCfe1 and *parA* gene-based assays are associated with considerable specificity failures and, consequently, these targets are unsuitable for *Cfv* identification when used solely. However, the detection of ISCfe1 or *parA* may be used as part of a diagnostic strategy, with validation of positive results to *parA* or ISCfe1 with previous or subsequent *C. fetus* detection assays. This strategy overcome the specificity failures associated to ISCfe1 and *parA* detection assays found in this study. Still, as described in previous studies [22, 23, 31], these molecular targets were sporadically found in *Cff* isolates, leading to false positive results even with the above diagnostic strategy. However, the specificity of these methods is expected to be higher if applied to bulls from farms with reproductive failure, where other causes were ruled out, as described previously [18].

## Conclusion

The results of this study have major implications in the molecular diagnosis of BGC, invalidating the use of ISCfe1 and *parA* as sole targets for *Cfv* identification. Diagnosis of BGC based solely on these genomic targets originates false positive results, which may lead to the unnecessary treatment or culling of animals. The combined use of *Cfv* and *C. fetus* detection assays should be encouraged as part of the BGC diagnostic strategy, in order to improve the specificity of molecular diagnostic methods. This study also described, for the first time, the presence of the *C. fetus* GI in another *Campylobacter* species inhabitant of the bull’s preputial mucosa, evidencing that this genomic element can be transferred to other C*ampylobacter* species. A highly homologous sequence of ISCfe1, previously considered a promising *Cfv* molecular marker, and the *parA* gene, were also found in another *Campylobacter* species. This evidences the horizontal gene transfer of current diagnostic *Cfv* molecular targets to other *Campylobacter* species and prompts for the development of reliable molecular diagnostic tools for BGC diagnosis.

## Methods

### Samples and DNA extraction

This observational study considered samples collected by certified veterinarians, within a bull breeding soundness examination, for diagnostic purposes.The number of tested bulls (*n* = 308) and herds (*n* = 61) is a representative sampling subset of the province of Alentejo, which represents the main beef cattle production area in Portugal, accounting over 10.000 beef cows of several breeds, in natural mating, distributed in the different regions of the province. Herds had unknown sanitary status for BGC, although some showed clinical features compatible with the disease (low breeding season fertility, extended time from bull introduction to conception depicted from calving records, embryo-fetal mortality observed at pregnancy diagnosis). Preputial samples were collected using a scraping/washing technique [32] and were sent to the laboratory under refrigeration conditions. Total DNA extraction was performed using the DNeasy Blood and Tissue Kit (Qiagen), according to the manufacturer’s instructions. Extracted DNA was quantified in a Nanodrop 2000C spectrophotometer (Thermo Scientific) and stored at − 20 °C until use.

### Real time PCR assays

Samples were tested by real-time PCR assays directed to subspecies (*Cfv*) and species (*C. fetus*) molecular targets. Detection at the subspecies level (*Cfv*) was performed using four assays, of which two targeting the *parA* gene (parA-A and parA-B assays) and the other two targeting the insertion element ISCfe1 (ISC-A and ISC-B assays). These pairs of assays, although directed to the same molecular target, detect different nucleotide regions as shown in Table 4. Additionally, two assays were performed for *C. fetus* detection, including an assay targeting the *nahE* gene and a commercial real-time PCR Kit (VetMAX *C. fetus* kit, Life Technologies). These two assays are designed to amplify sequences common to both *C. fetus* subspecies (*Cfv* and *Cff*), and, consequently, do not allow the identification at the subspecies level.

**Table 4:** Real-time PCR assays used for *Cfv* detection

Assay	Target	Nucleotide Region ^a^	Reference
ISC-A	ISCfe1: *tnpB* gene	764–843	This study
ISC-B	ISCfe1: *tnpA* gene	567–626	[10]
parA-A	*parA* gene	321–406	[13], with modifications
parA-B	*parA* gene	84–161	This study

*n*: number of samples tested; ^a^ Nucleotide region of ISCfe1 and *parA* sequences with NCBI accession numbers AM260752.1 and CP043435.1: c1229121–1,228,459, respectively

**Fig. 3:**
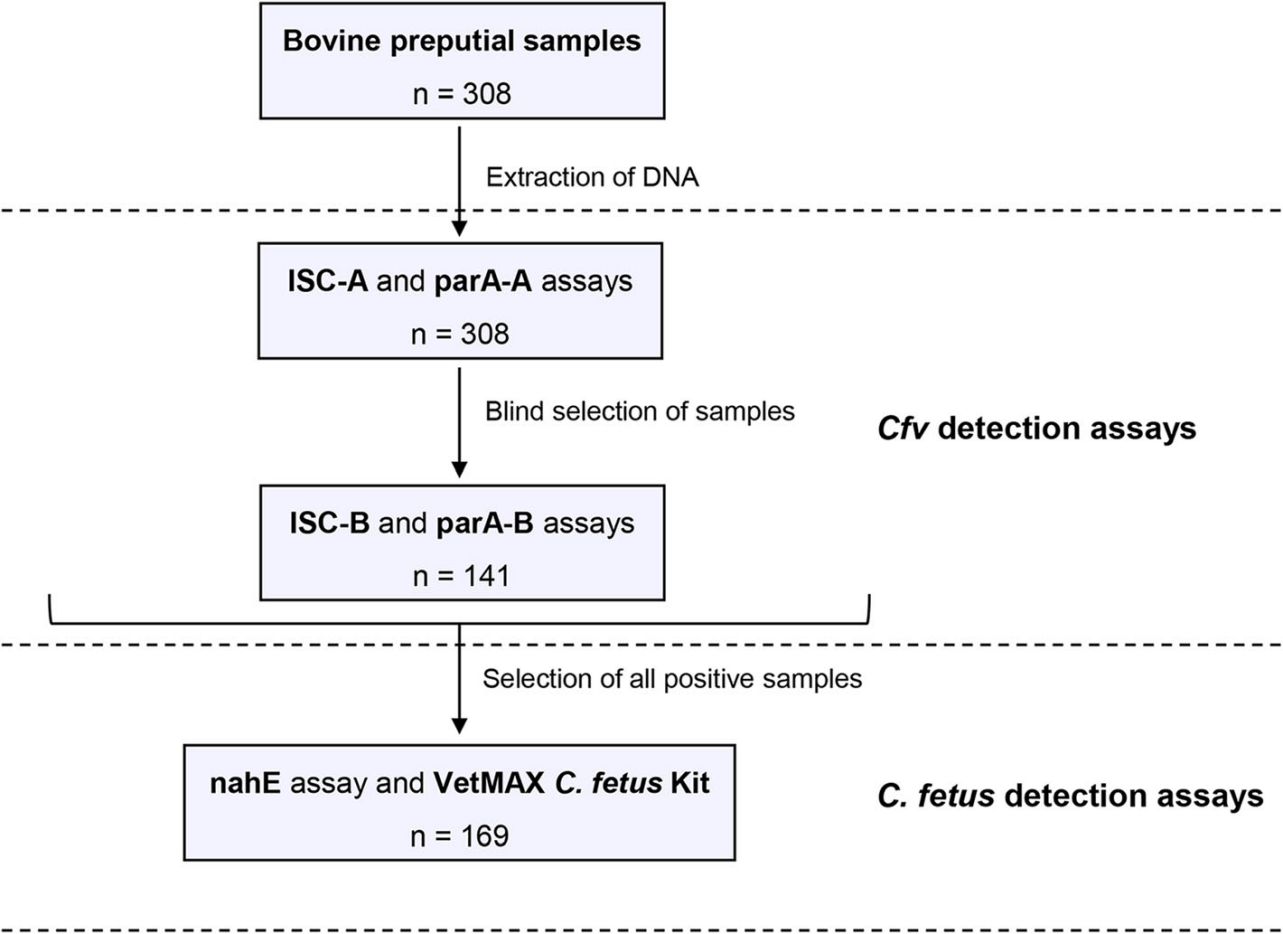
Flow chart of the sampling procedure for *Cfv* and *C. fetus* detection assays

The flow chart of the sampling procedure for *Cfv* and *C. fetus* detection assays is schematically represented in Fig. 3. Briefly, all samples (*n* = 308) were tested with ISC-A and parA-A assays. In addition, a subset of these samples (*n* = 141) was blindly selected to be tested with ISC-B and parA-B assays. Detection of *C. fetus* specific targets was performed in all ISCfe1 and/or *parA* gene positive samples (*n* = 169).

For all assays, a PCR positive control was used in every experiment, which was prepared by adding 100 copies of *Cfv* genomic DNA from strain NCTC 10354 to 25 ng of DNA extracted from a *Cfv*-negative preputial sample to simulate a *Cfv*-positive preputial sample.

#### Detection of ISCfe1 insertion sequence: ISC-A and ISC-B assays

For the detection of the insertion element ISCfe1 two different Taqman MGB probe-based assays were used (ISC-A and ISC-B assays; Table 4). The ISC-A assay was developed to detect the *tnpB* gene of ISCfe1 (Table 4). The primers and probe (Additional file 2) were designed to target a 109 bp sequence using Primer Express software v2.0 and specificity was assessed using BLAST search in the NCBI database. PCR reactions were performed in duplicate, in 20 μL mixtures containing 1x SensiFAST probe Hi-ROX mastermix (Bioline Reagents Ltd), 400 nM of each primer, 100 nM of Taqman MGB probe and 25 ng of total DNA. Amplifications were performed on a StepOnePlus system (Applied Biosystems), using the following thermal cycle conditions: 2 min incubation step at 50 °C, denaturation for 4 min at 95 °C, followed by 35 cycles of 10 s at 95 °C and 30 s at 60 °C. The ISC-B assay targets the *tnpA* gene of ISCfe1, using previously published primers and probe [10]. PCR reaction mixtures and thermal cycle conditions were as previously described for ISC-A. Samples with a Ct < 35 were considered positive.

#### Detection of parA gene: parA-A and parA-B assays

The detection of *parA* gene also used two different Taqman MGB probe assays (parA-A and parA-B assays; Table 4). The parA-A assay is a real-time PCR assay previously described [13], with minor modifications (Additional file 2). The reverse primer was modified according to sequencing data of PCR products amplified with primers VENSF/VENSR [12] in the bovine preputial samples. All reactions were carried out in 20 μL reaction volume, with 900 nM of each primer, 250 nM of Taqman MGB probe, 1x SensiFAST probe Hi-ROX mastermix and 25 ng of DNA. Amplifications were performed on a Step One Plus System, using the thermal cycle conditions previously described [13]. The parA-B assay was designed to amplify a different sequence of the *parA* gene with 78 bp (Table 4). The primers and probe (Additional file 2) were designed using Primer Express software v2.0 and specificity was assessed using BLAST search in the NCBI database. PCR reaction mixtures and thermal cycle conditions were the same as described for ISC-A assay. Samples with a Ct < 35 in parA-A or parA-B assays were considered positive.

#### Detection of C. fetus species-specific targets: nahE and VetMAX C. fetus kit

*C. fetus* detection was performed using a real-time PCR assay targeting the *nahE* gene and a commercial diagnostic kit (VetMAX *C. fetus* kit, Life Technologies) in all samples positive to ISCfe1 (ISC-A and/or ISC-B assays) and/or *parA* gene (parA-A and/or parA-B assay). The *nahE* gene was detected using previously described primers and probe [10], with the conditions described above for ISCfe1 amplification. Samples with a Ct < 35 were considered positive*.* Samples were also tested with VetMAX *C. fetus* kit following the instructions and validation criteria recommended by the manufacturer.

#### Real-time PCR assays’ performance: linearity, amplification efficiency and reproducibility

The analytical performance of the real-time PCR assays was assessed in DNA extracted from preputial samples, spiked with genomic DNA from *Cfv* strain NCTC 10354. Standards were prepared using DNA from *Cfv* NCTC 10354, previously extracted with Qiagen DNeasy Blood and Tissue kit (Qiagen). The genomic DNA was quantified using a Nanodrop 2000C spectrophotometer and the number of *Cfv* genome copies was determined based on the strain’s genome size (1,874,244 bp), according to the formula: Number of copies = (DNA concentration (ng/μL) x [6.022 × 10^23^]) / (length of template (bp) x [1 × 10^9^] × 650). Ten-fold dilutions were performed from 1.25 × 10^6^ to 1.25 × 10^2^ genome copies/μL. Then, DNA preputial samples of three bulls, previously tested as negative in the PCR assays under study, were spiked with ten-fold dilutions of *Cfv* NCTC 10354, representing 10^1^ to 10^5^ genome copies per 25 ng of preputial sample DNA in 2 μL of template (PCR reaction). These three sets of standards were aliquoted and stored at − 20 °C until use. Three independent experiments were performed for each real-time PCR assay, one for each set of standards, all tested with two replicate wells per dilution. The performance of the real-time PCR assays was evaluated based on the linearity (*r*^2^), amplification efficiency (E) and reproducibility. The amplification efficiency was estimated based on the slope of the standard curve, using the formula: E = 10 ^(− 1/slope)^ -1. The reproducibility was evaluated using the inter-assay and intra-assay coefficients of variation (CV) for each dilution.

### Detection of genomic island genes in bovine preputial samples

The presence of GI genes formerly considered *Cfv*-specific, which include type 4 secretion system-coding genes (*virB2-virB11*/*virD4* genes), was assessed by conventional PCR. This *Cfv*-associated genomic island may also include the *parA* gene and *fic* genes. Amplification of *virB9* and *virB11* was performed with previously described primers [11]. Primers for amplification of *fic1* and *fic2* (Additional file 2) were designed with Primer-BLAST [33] using the sequence of *Cfv* strain NCTC 10354 as a reference. These assays for *fic1*, *fic2*, *virB9* and *virB11* gene detection were performed in 49 samples, from which 30 *parA* negative and 19 *parA* positive in the parA-A assay. PCR reactions were carried out in 25 μL mixtures containing 0.4 μM of each primer, 400 μM of each dNTP (4you4 dNTP Mix, Bioron), 1 x reaction buffer (Complete reaction buffer, Bioron), 2 units of DFS-Taq DNA polymerase (Bioron) and 150 ng of DNA. Amplifications were performed in a Doppio thermal cycler (VWR) using the following conditions: 3 min at 94 °C, followed by 35 cycles of 94 °C for 30 s, annealing temperature for 30 s, and 72 °C for 1 min, with a final extension step of 5 min at 72 °C. The annealing temperatures selected for amplification of *fic1/fic2*, *virB9* and *virB11* were 57 °C, 53 °C and 56 °C, respectively. Amplification products were detected by electrophoresis on a 1.5% agarose gel stained with ethidium bromide and bands were visualized in a ChemiDoc XRS+ System (Biorad).

### Statistical analysis

Statistical data analysis was performed using IBM SPSS Statistics for Windows, version 26.0 (IBM Corporation). To evaluate inter and intra-assay reproducibility of real-time PCR assays, the coefficient of variation of the Ct was calculated as follows: % CV = (standard deviation Ct / mean Ct) × 100. Agreement between results of different real-time PCR assays was evaluated using the Cohen’s Kappa coefficient. Additionally, results were analysed using McNemar’s test for testing the null hypothesis that methods were equally likely to identify samples as positive or negative. The presence of association between the detection of GI-associated genes and the detection of the *parA* gene was assessed using the phi (φ) coefficient. Values of *P* < 0.05 were considered statistically significant.

## Supplementary Information

**Additional file 1.** Agreement between assays directed towards the same molecular target. (a) Agreement between parA-A and parA-B assays, (b) Agreement between ISC-A and ISC-B assays.

**Additional file 2.** Primers and probes used in real time PCR assays.

## Data Availability

The Whole Genome Shotgun project of *Campylobacter portucalensis* is deposited at DDBJ/ENA/GenBank under the accession number VWSJ00000000.1, which includes the insertion sequence (Contig 29, VWSJ01000029.1) and the genomic island (Contig 27, VWSJ01000027.1). Other datasets used and/or analysed during the current study are available from the corresponding author upon request.

## Abbreviations

BGC: Bovine Genital Campylobacteriosis
*Cff*: *Campylobacter fetus* subsp. *fetus*
*Cfv*: *Campylobacter fetus* subsp. *venerealis*
Ct: Threshold cycle
CV: Coefficient of variation
GI: Genomic island
