# Supplementary material for: Assessment of Campylobacter fetus subsp. venerealis molecular diagnosis using clinical samples of bulls
Source: BMC Vet Res. 2020 Oct 29;16:410. doi: 10.1186/s12917-020-02634-7 (PMC7596931; doi:10.1186/s12917-020-02634-7)
Supplement: Supplementary file 1 — Additional file 1. Agreement between assays directed towards the same molecular target. (a) Agreement between parA-A and parA-B assays, (b) Agreement between ISC-A and ISC-B assays. [file 12917_2020_2634_MOESM1_ESM.pdf]

Additional file 1. Agreement between assays directed towards the same molecular target

(a) Agreement between parA-A and parA-B assays.

parA-A	parA-B		Total
	Positive	Negative	
Positive	11 (7.8%)	29 (20.6%)	40 (28.4%)
Negative	0 (0 %)	101 (71.6%)	101 (71.6 %)
Total	11 (7.8%)	130 (92.2%)	141

(b) Agreement between ISC-A and ISC-B assays.

ISC-A	ISC-B		Total
	Positive	Negative	
Positive	24 (17.0%)	59 (41.8 %)	83 (58.9%)
Negative	0 (0%)	58 (41.1%)	58 (41.1%)
Total	24 (17.0%)	117 (83.0%)	141
