# Supplementary material for: Assessment of Campylobacter fetus subsp. venerealis molecular diagnosis using clinical samples of bulls
Source: BMC Vet Res. 2020 Oct 29;16:410. doi: 10.1186/s12917-020-02634-7 (PMC7596931; doi:10.1186/s12917-020-02634-7)
Supplement: Supplementary file 2 — Additional file 2. Primers and probes used in real time PCR assays. [file 12917_2020_2634_MOESM2_ESM.pdf]

Additional file 2. Primers and probes used in real time PCR assays

Target (Assay)	Designation	Function	Sequence (5'-3')	Reference
ISCfe1 (ISC-A)	ISC-A_F	Forward primer	AAACCAAACAATAAAGCAATCACTCA	This study
	ISC-A_R	Reverse primer	ACACCTTGCTTATAATACTCTTGCCATT	
	ISC-A_P	Probe	**FAM**-TTGGCTGTTCTCGTTTAG-**MGB-NFQ**	
ISCfe1 (ISC-B)	ISC1-F	Forward primer	AGGCGAAGAGAATGTTAAATTTGAA	van der Graaf-van Bloois et al. (2013)
	ISC1-R	Reverse primer	CCATAAAGCCTAGCTGAAAAAACTG	
	ISC_P	Probe	**FAM**-CCAAAGATGTCTTAGAAATA-**MGB-NFQ**	
parA (parA-A)	CFVF	Forward primer	CCCAGTTATCCCAAGCGATCT	McMillen et al. (2006)
	CFVR	Reverse primer	CG**T**T**G**GGATT**G**TAAATTTTAGCTTG**T**T *	
	CFVP1	Probe	**FAM**-CATGTTATTTAATACCGCAA-**MGB-NFQ**	
parA (parA-B)	parA-B_F	Forward primer	CGGCGATGATACGCTTTTAGT	This study
	parA-B_R	Reverse primer	GAGCTATCTGCTCTAATGTCCGTAAAT	
	parA-B_P	Probe	**VIC**-CGATCCACAAAGAAGTAT-**MGB-NFQ**	
fic1	fic1_F	Forward primer	CGATGTCATTGTTGCGCAGT	This study
	fic1_R	Reverse primer	AGCAACTAGCGAGCGTGAAT	
fic2	fic2_F	Forward primer	TGACCTTTTGGGCTGTTTGG	This study
	fic2_R	Reverse primer	GAGCTTGGCGATATGCTGGA	
virB9	nC1165g2F	Forward primer	TGACAAAGATGAGCGGATAG	Moolhuijzen et al. (2009)
	nC1165g4R	Reverse primer	TACCTGTTCGCCGTTTTC	
virB11	nC1165g4F	Forward primer	AGGACACAAATGGTAACTGG	Moolhuijzen et al. (2009)
	nC1165g4R	Reverse primer	GATTGTATAGCGGACTTTGC	
nahE	nahE-F	Forward primer	TGTTATGGTGATCAAAATAGCTGTTG	van der Graaf-van Bloois et al. (2013)
	nahE-R	Reverse primer	GAGCTGTTTTTATGGCTACTCTTTTTTTA	
	nahE-P	Probe	**VIC**-TGTATATGCACTTTTAGCAACTT-**MGB-NFQ**	

* Nucleotide modifications in the primer sequence originally described by McMillen et al. (2006) are underlined.
